# Heterogeneity Analysis of HBeAg-Positive Chronic Hepatitis B Patients with Ultra-High Viral Load (HBV DNA ≥ 7.0 log_10_ IU/mL)

**DOI:** 10.3390/jcm15062164

**Published:** 2026-03-12

**Authors:** Guifeng Li, Rong Ren, Jie Liu, Jia Li

**Affiliations:** 1 The Second People’s Hospital Affiliated to Tianjin Medical University, Tianjin 300211, China; a280156100@gmail.com (G.L.); tiderr@163.com (R.R.); 2 Tianjin Second People’s Hospital, Tianjin 300192, China; liujie_0802@163.com

**Keywords:** HBeAg-positive chronic hepatitis B, ultra-high HBV DNA, HBV DNA ≥ 7.0 log10 IU/mL, retrospective study, phenotypic heterogeneity, cluster analysis, transient elastography

## Abstract

**Background/Objectives**: HBeAg-positive chronic hepatitis B (CHB) patients with very high viral replication are often clinically considered a homogeneous, low-risk population. However, substantial biochemical, virological, and fibrosis-related heterogeneity may exist. This study aimed to characterize this heterogeneity in treatment-naive, HBeAg-positive CHB patients with ultra-high viral loads (HBV DNA ≥ 7.0 log_10_ IU/mL). Furthermore, we sought to identify predictors of significant fibrosis and detect clinically relevant discordant phenotypes, such as silent disease progression despite normal alanine aminotransferase (ALT) levels. **Methods**: This single-center, retrospective, cross-sectional study analyzed consecutively screened eligible patients. A liver stiffness measurement (LSM, kPa) and controlled attenuation parameter (CAP, dB/m) were obtained via transient elastography. Significant fibrosis was defined as an LSM ≥ 7.0 kPa. Statistical evaluations included Spearman’s correlation, multivariable regression, ALT-LSM stratification, and K-means clustering. **Results**: Among 413 included patients, age and aspartate aminotransferase (AST) emerged as independent risk factors for significant fibrosis, whereas log10 HBV DNA and log10 HBsAg were independent negative predictors. Patients with HBsAg ≥ 25,000 IU/mL exhibited significantly lower LSM values than those with lower HBsAg levels. Notably, 18.4% of patients with strictly normal ALT (≤40 U/L) presented with an LSM ≥ 7.0 kPa, indicating silent progression. Cluster analysis further identified two distinct patient phenotypes characterized by differing age, ALT, viral load, and fibrosis profiles. **Conclusions**: An ultra-high viral load in HBeAg-positive CHB does not guarantee a uniformly benign clinical state. By quantifying biochemical, virological, and fibrotic heterogeneity, this study highlights a critical subgroup with silent fibrosis progression that risks being overlooked by ALT-based assessments alone.

## 1. Introduction

Understanding the natural history and phase classification of chronic hepatitis B (CHB) remains challenging, as the standard phase structure fails to adequately reflect the dynamic interactions between host and virus in real-world scenarios [1]. Typically, high serum HBV DNA levels are associated with the HBeAg-positive high-replication phase, often described as “immune tolerance.” However, patients within this category frequently exhibit significant biochemical variability, with ALT levels fluctuating between normal and elevated ranges. This variability indicates diverse immune activity rather than a stable state [2].

Current guidelines offer structured thresholds and algorithms to guide treatment decisions. However, these decisions become challenging for patients who do not fit the “classical” profile, such as those with “silent” conditions characterized by high viral replication and normal ALT levels, yet who still experience clinically significant liver injury. Recent evidence and guidelines advocate for simplifying care and adopting more targeted, risk-strategic approaches. This includes using noninvasive tests (NITs) for fibrosis assessment instead of relying solely on ALT-based definitions [3,4].

Current data indicate that the HBeAg-positive high-replication period is not a static immune-tolerant phase; instead, it encompasses various immune states with differing levels of inflammation and fibrosis activity. Traditionally, it was believed that HBeAg-positive patients with normal ALT levels were in an immune-tolerant phase, and thus, antiviral therapy was not recommended to avoid potential long-term adverse effects [5,6,7]. However, recent population-based studies and meta-analyses have revealed a significant risk of hepatocellular carcinoma (HCC) in patients who remain in indeterminate or gray-zone phases. These studies have also demonstrated that antiviral therapy can significantly reduce this risk in such groups [2].

A normal ALT level does not necessarily indicate the absence of disease. Both biopsy and real-world studies reveal that many CHB patients with normal ALT levels still experience significant necroinflammation and/or fibrosis. This supports the concept of “silent” liver damage accumulating despite seemingly stable biochemistry [8,9,10]. This biochemical–pathological mismatch likely results in misclassification in current clinical practice. First, normal ALT levels can conceal significant fibrosis, potentially delaying treatment for patients who are already progressing. Second, transient elastography-derived LSM might be influenced by inflammation, leading to an overestimation of fibrosis stage during acute necroinflammation [11].

These observations highlight the need for a more precise characterization of heterogeneity within the HBeAg-positive, ultra-high-viremia population. The novelty of the present study is not merely to restate that variability exists within HBeAg-positive CHB, but to quantify clinically relevant heterogeneity across biochemical (ALT/AST), virological (HBV DNA/HBsAg/HBeAg), and fibrosis-related (LSM) dimensions in a narrowly defined cohort with HBV DNA ≥ 10^7^ IU/mL. In addition, we aimed to identify independent predictors of significant fibrosis, describe biochemical–virological discordance, and detect phenotypic subgroups with silent progression that may be overlooked by conventional ALT-centered assessment [1,3,4,12].

## 2. Materials and Methods

### 2.1. Study Design and Participants

This was a single-center retrospective observational study conducted at Tianjin Second People’s Hospital from January 2010 to January 2025. This manuscript reports a baseline cross-sectional analysis within that retrospective cohort to characterize heterogeneity at the time of clinical evaluation. Ultra-high viral load was predefined as serum HBV DNA ≥ 7.0 log_10_ IU/mL (≥10^7^ IU/mL). Eligible participants were treatment-naïve adults (≥18 years) with chronic HBV infection (HBsAg positivity for ≥6 months), HBeAg positivity, ultra-high HBV DNA, and available baseline clinical/laboratory data plus reliable transient elastography. Patients with prior antiviral therapy, coinfection (e.g., HCV/HIV), other chronic liver diseases, overt hepatic decompensation, or missing key data were excluded according to the original protocol.

The Ethics Committee of Tianjin Second People’s Hospital reviewed and approved the study, ensuring compliance with the Declaration of Helsinki. The ethical approval number is Tianjin Second People’s Hospital Ethics Review No. [2024] 70. Given that the study utilized existing clinical data, the ethics committee waived the requirement for written informed consent from the patients. Initially, 1050 patients with chronic hepatitis B and high viral loads were screened. Out of these, 413 had complete liver stiffness measurements (LSM) and relevant covariates, which were included in the analysis.

### 2.2. Clinical and Laboratory Parameters

Baseline variables included age, sex, alanine aminotransferase (ALT), aspartate aminotransferase (AST), alpha-fetoprotein (AFP), quantitative HBsAg, quantitative HBeAg, and HBV DNA. For the primary analysis, the upper limit of normal (ULN) for ALT was defined as 40 U/L. Quantitative HBsAg and HBeAg (IU/mL) were measured using the Roche Cobas e601 platform, and HBV DNA was quantified by real-time PCR. Data on HBV genotype distribution and precore/basal core promoter mutations were not routinely available in this retrospective dataset and therefore could not be incorporated into the present analyses.

Transient elastography was performed using FibroScan 502 Touch (Echosens, Paris, France) by experienced operators. Liver stiffness measurement (LSM, kPa) was used as a non-invasive surrogate of fibrosis burden, and controlled attenuation parameter (CAP, dB/m) was used as a non-invasive surrogate of hepatic steatosis. At least 10 valid measurements were obtained per patient, and reliability criteria were applied according to routine practice standards. Significant fibrosis was operationally defined as LSM ≥ 7.0 kPa based on published chronic hepatitis B elastography literature and guideline-supported non-invasive fibrosis assessment frameworks; sensitivity analyses using higher thresholds (e.g., 8.0, 9.5, and 12.0 kPa) were also considered to test robustness and to approximate more advanced fibrosis/cirrhosis-risk strata.

### 2.3. Statistical Analysis

Statistical analyses were conducted using R software (version 4.4.1). Normality was assessed using the Shapiro–Wilk test. Continuous variables are presented as mean ± standard deviation (SD) or median (interquartile range [IQR]) according to distribution, and categorical variables are presented as counts and percentages. HBV DNA, HBsAg, and HBeAg values were log10-transformed when analyzed as continuous predictors.

Continuous variables were compared using the independent Student’s *t*-test or Mann–Whitney U test as appropriate, and categorical variables were compared using the chi-squared test or Fisher’s exact test. To characterize heterogeneity, prespecified Spearman correlation analyses were performed among fibrosis-related markers (LSM), biochemical markers (ALT, AST, AFP), virological markers (HBV DNA, HBsAg, HBeAg), CAP, and age. *p*-values from correlation analyses were adjusted for multiple comparisons using false-discovery-rate control.

To identify independent predictors of LSM as a continuous outcome, multivariable linear regression was performed. Independent predictors of significant fibrosis (LSM ≥ 7.0 kPa) were identified using multivariable logistic regression with forward stepwise selection. Candidate variables entered into the selection pool—age, sex, ALT, AST, CAP, log_10_ HBsAg, log_10_ HBV DNA, and AFP—were chosen on the basis of clinical relevance and univariable screening (*p* < 0.10); variables were retained in the final model at *p* < 0.05. Prior to model fitting, potential collinearity among covariates was evaluated by examining the correlation structure and computing variance inflation factors (VIFs), with a VIF exceeding 5 taken as evidence of meaningful collinearity. All tests were two-sided; *p* < 0.05 was considered statistically significant. Results are reported as beta coefficients for the linear model and as odds ratios with 95% confidence intervals for the logistic model.

Biochemical-fibrosis discordance (‘silent progression’) was estimated using two-dimensional stratification based on ALT (>40 U/L vs. ≤40 U/L) and LSM (≥7.0 kPa vs. <7.0 kPa). K-means clustering was applied to standardized key variables (age, ALT, LSM, CAP, HBsAg, and HBV DNA) to identify composite phenotypes, and the optimal number of clusters was selected using the silhouette coefficient.

## 3. Results

### 3.1. Baseline Characteristics

A total of 413 patients were included, comprising 217 males (52.5%) and 196 females (47.5%), with a mean age of 38.23 ± 9.60 years (range: 18–69 years). All participants were HBeAg-positive and had ultra-high viral load by study definition. The cohort showed substantial variability in biochemical and fibrosis-related indices despite similarly high viral replication. The median ALT was 59.00 U/L, and ALT variability was wide, with 65.3% of patients having ALT > 40 U/L, 38.8% > 80 U/L. Because liver biopsy was not performed systematically, fibrosis burden was characterized using LSM-based strata rather than histologic stage in the main analysis. HBV genotype and mutation data were unavailable in the routine retrospective dataset.

Gender-based analysis revealed that males had significantly higher levels of ALT, CAP, and LSM compared to females [ALT: 71.00 (30.00–123.00) vs. 49.00 (25.00–71.00) U/L; CAP: 231.00 (195.00–257.00) vs. 211.00 (195.00–257.00) dB/m; LSM: 6.50 (5.10–9.20) vs. 6.10 (5.10–9.20) kPa; all *p* < 0.05]. Detailed characteristics are summarized in Table 1. In supplementary analyses, significant clinical heterogeneity was observed across subgroups stratified by HBV DNA, age, ALT, and LSM. Specifically, lower HBV DNA levels and advanced age were associated with reduced viral/antigen levels but higher LSM. Conversely, patients in the higher ALT and LSM strata exhibited significantly elevated AST and AFP levels (all *p* < 0.05; Supplementary Tables S1–S4).

### 3.2. Correlation Analysis

A total of 413 HBeAg-positive patients with ultra-high viral loads were included in this study. The cohort comprised 217 males (52.5%) and 196 females (47.5%), with a mean age of 38.23 ± 9.60 years (range: 18–69 years). Despite uniformly high viral replication, the patients exhibited substantial heterogeneity in biochemical and fibrosis-related indices. Specifically, the median alanine aminotransferase (ALT) level was 59.00 U/L, with 65.3% and 38.8% of patients presenting with ALT > 40 U/L and >80 U/L, respectively. As liver biopsies, HBV genotypes, and mutation data were unavailable in this routine retrospective dataset, fibrosis burden was non-invasively evaluated using liver stiffness measurement (LSM) strata.

**Figure 1:**
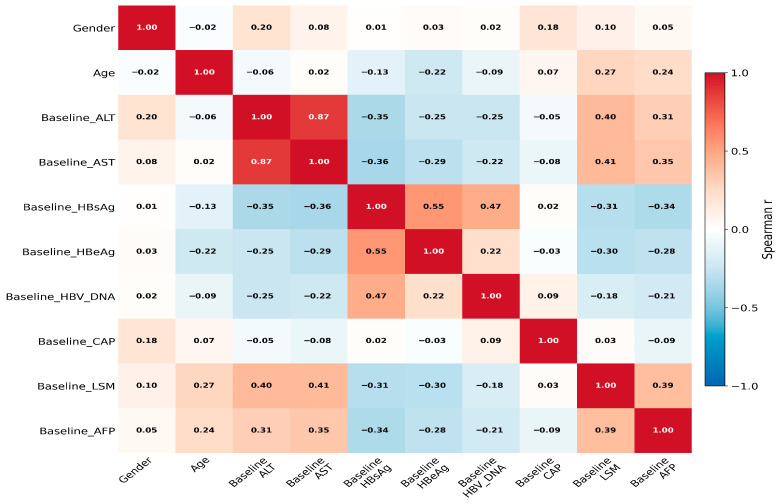
Spearman Correlation Heatmap of Clinical and Virological Indicators.

Gender-stratified analysis revealed that male patients had significantly higher levels of ALT, controlled attenuation parameter (CAP), and LSM compared to females [ALT: 71.00 (30.00–123.00) vs. 49.00 (25.00–71.00) U/L; CAP: 231.00 (195.00–257.00) vs. 211.00 (195.00–257.00) dB/m; LSM: 6.50 (5.10–9.20) vs. 6.10 (5.10–9.20) kPa; all *p* < 0.05] (Figure 1). The relationships among ALT, age, HBV DNA, and fibrosis-related markers are further illustrated in Supplementary Figure S1. Detailed baseline characteristics are summarized in Table 1.

### 3.3. Identification of Independent Predictors for LSM

We performed a multivariable linear regression analysis to identify independent predictors of continuous LSM. As detailed in Table 2, age demonstrated a significant positive association with LSM (β = 0.133, 95% CI: 0.031–0.235, *p* = 0.011), whereas baseline log_10_ HBsAg was identified as an independent negative predictor (β = −3.063, 95% CI: −5.769 to −0.357, *p* = 0.027). AST levels showed a positive but non-significant trend with LSM (β = 0.011, *p* = 0.073). No significant associations were found for other clinical variables, including gender, ALT, CAP, log_10_ HBV DNA, and AFP (all *p* > 0.05).

### 3.4. Risk Factors for Significant Liver Fibrosis

**Figure 2:**
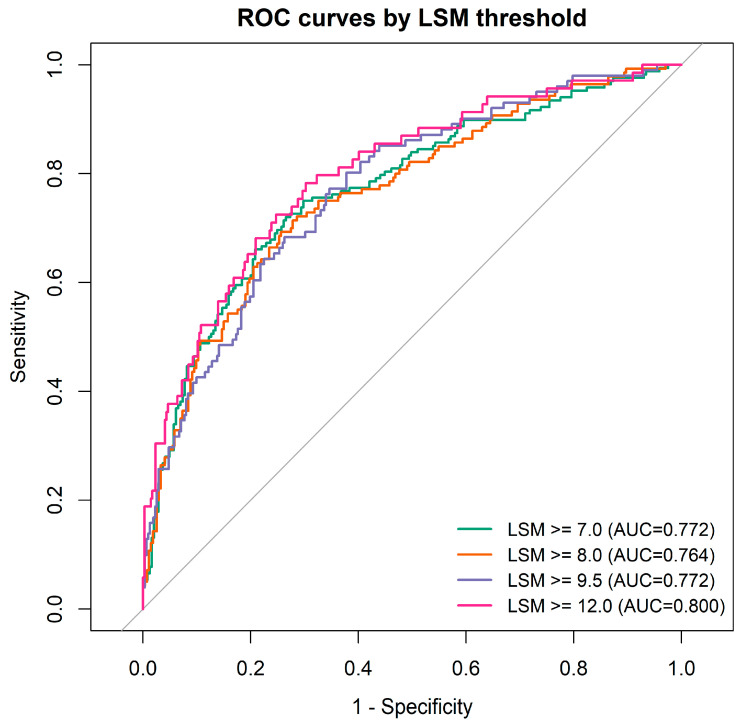
ROC Curve for Predicting Significant Fibrosis.

A multivariable logistic regression analysis was conducted using an LSM ≥ 7.0 kPa to define significant fibrosis (Table 3; ROC analysis in Figure 2). Advanced age and elevated AST independently predicted an increased risk of significant fibrosis (both *p* < 0.05), while log_10_ HBV DNA and log_10_ HBsAg served as independent negative predictors. Gender, ALT, and CAP lost statistical significance after multivariable adjustment. The independent association of AST highlights its potential to provide complementary clinical insights in patients with discordant profiles (e.g., normal ALT but high LSM), pending further prospective validation.

### 3.5. Two-Dimensional Stratification of ALT and LSM

Patients were divided into four groups according to ALT (>40 U/L vs. ≤40 U/L) and LSM (≥7.0 kPa vs. <7.0 kPa). Among patients with normal ALT (≤40 U/L), 26 of 141 (18.440%) had LSM ≥ 7.0 kPa and were classified as the ‘silent progression (SP)’ subgroup. Among patients with elevated ALT (>40 U/L), 142 of 272 (52.206%) had LSM ≥ 7.0 kPa. This two-dimensional stratification shows that ALT alone is insufficient to exclude clinically meaningful fibrosis in this population (Table 4).

### 3.6. Impact of HBsAg on LSM

Patients were stratified into two groups based on the HBsAg threshold of 25,000 IU/mL. As shown in Table 5, patients with high HBsAg levels (≥25,000 IU/mL, *n* = 230) exhibited significantly lower liver stiffness compared to those with lower HBsAg levels (*n* = 183) [Median: 6.00 (IQR: 4.80–7.55) vs. 7.60 (IQR: 5.70–12.40) kPa; *p* < 0.001]. These findings further support the negative association between HBsAg quantification and liver fibrosis in this high-viral-load cohort.

### 3.7. Cluster Analysis

**Figure 3:**
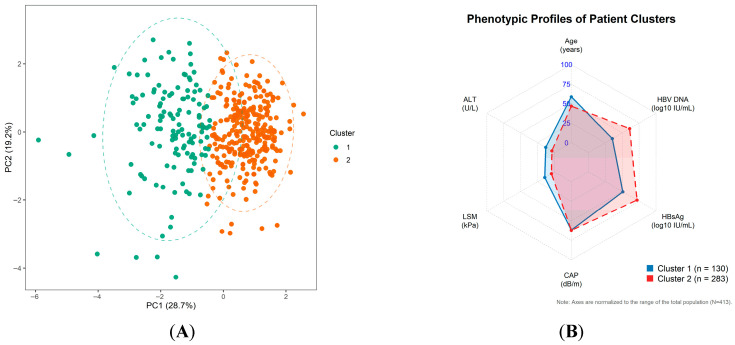
(**A**) PCA scatter plot of K-means clustering. (**B**) Radar Chart of K-means Clustering.

K-means clustering was applied to standardized key variables (age, ALT, LSM, CAP, HBsAg, and HBV DNA). The highest silhouette coefficient (0.286) was observed at k = 2, indicating two composite phenotypes. Cluster 1 (*n* = 130) was characterized by older age, markedly higher ALT and LSM, and relatively lower HBV DNA/HBsAg, representing an inflammation/fibrosis-dominant phenotype (Figure 3). Cluster 2 (*n* = 283) was younger and showed lower ALT and LSM with higher virological markers, representing a virus-dominant/low-stiffness phenotype. The substantial ALT difference between clusters (Table 6) further supports clinically meaningful biochemical heterogeneity within this seemingly uniform high-viremia population.

## 4. Discussion

### 4.1. Phenotypic Heterogeneity Within the Ultra-High-Viremia Spectrum

The present study demonstrates that HBeAg-positive CHB patients with ultra-high viral load (HBV DNA ≥ 10^7^ IU/mL) cannot be treated as a clinically uniform group. Using a combination of correlation analysis, multivariable regression, two-dimensional ALT-LSM stratification, and unsupervised clustering, we identified substantial heterogeneity across biochemical, virological, and fibrosis-related dimensions within this narrowly defined cohort. Notably, 18.4% of patients with normal ALT (≤40 U/L) had LSM ≥ 7.0 kPa, meeting the operational criterion for significant fibrosis—a finding consistent with the concept of silent liver injury accruing in the apparent absence of biochemical activity [6,7,10,13,14]. These observations suggest that ultra-high viral load alone is insufficient to define a patient’s fibrosis risk, and that ALT-centric assessment may underestimate disease burden in a clinically meaningful subgroup.

The K-means clustering analysis (Figure 3) provides complementary, multidimensional support for this conclusion. The PCA scatter plot (Figure 3A) reveals spatial separation between the two identified clusters in the reduced-dimensionality feature space, indicating that patients can be distinguished into two phenotypically distinct subgroups despite sharing the same ultra-high viremia criterion. The radar chart (Figure 3B) illustrates that Cluster 1 is characterised by relatively higher ALT and LSM alongside lower viral markers, consistent with a host inflammation- and fibrosis-dominant phenotype, while Cluster 2 displays higher HBV DNA and HBsAg with lower liver stiffness and transaminase values, consistent with a virus-dominant, low-inflammatory phenotype. The silhouette coefficient at k = 2 (0.286), while modest, suggests that a two-cluster solution offers the most interpretable structure in this dataset, though the degree of overlap visible in the PCA plot indicates that these phenotypes represent tendencies along a continuum rather than discrete biological categories. Taken together, these findings are consistent with the view that the so-called immune-tolerant phase encompasses a range of host–virus interaction states rather than a single homogeneous condition—though this interpretation requires prospective longitudinal validation to establish its clinical and prognostic implications.

### 4.2. Mechanistic Context: Immune Regulation in High-Viremia CHB

The mechanistic basis for phenotypic heterogeneity within the high-replication phase has been explored in a growing body of experimental and clinical literature, which we summarise here as contextual background for interpreting our observational findings.

Published data suggest that the immunotolerant state is primarily characterised by suppressed or functionally depleted virus-specific immune responses rather than simply an absence of immune recognition [12,15,16]. Sustained exposure to high concentrations of HBeAg and HBsAg has been associated with HBV-specific T-cell exhaustion, evidenced by upregulation of inhibitory receptors such as PD-1 and Tim-3 and expansion of regulatory immune populations—effects that are reportedly most pronounced in the hepatic microenvironment [16,17,18]. Experimental models further suggest a hepatocyte antigen burden-dependent mechanism: when the majority of hepatocytes carry high viral antigen loads, infiltrating HBV-specific CD8^+^ T cells may undergo functional impairment or deletion, thereby limiting immune-mediated hepatocyte injury; when antigen density falls below a certain threshold, residual T-cell effector activity may permit partial immune control, but at the cost of necroinflammation and fibrogenic signalling [19,20,21]. These mechanistic models, if applicable to the clinical setting, would offer a plausible explanation for the inverse association between viral load and fibrosis burden observed both in the present cohort and in prior studies [3,22,23]. It should be emphasised, however, that our study was not designed to test these mechanisms directly, and the immunological interpretations offered here are grounded in the cited experimental literature rather than in immunological data generated from this cohort.

With respect to disease phase transitions, longitudinal data from untreated immune-tolerant cohorts indicate that a substantial minority of patients progress to the immune-active phase over medium-term follow-up. In one cohort of 109 immune-tolerant patients followed for a mean of 2.17 years, 30.3% transitioned to immune-active disease [7]. Such data suggest that the high-replication phase is not necessarily stable over time, and that individual patients may progress at different rates depending on factors that remain incompletely understood, including age, immune maturation, and the possible emergence of viral escape variants [15,19]. Our cross-sectional data cannot address transition rates or temporal dynamics, but the fibrosis heterogeneity we observed at baseline is at least consistent with the hypothesis that some patients have already experienced periods of intermittent immune activity prior to evaluation.

### 4.3. Limitations of ALT-Based Assessment and the Role of Non-Invasive Fibrosis Testing

The present data add to a body of evidence indicating that ALT alone provides an incomplete picture of liver disease severity in CHB. A number of factors may contribute to this discordance. First, the definition of the normal ALT range varies across guidelines—the AASLD recommends 35 U/L for men and 25 U/L for women, whereas EASL and most Asia-Pacific guidelines retain 40 U/L—meaning that patients with ALT in the high-normal range (30–40 U/L) may be classified differently depending on the reference applied [3,24,25,26,27]. Second, ALT reflects hepatocyte injury at a given time point and may not reliably detect mild or slowly progressive disease activity [10,28,29]. Consistent with these concerns, Lai et al. found significant fibrosis or inflammation on biopsy in 37% of CHB patients with persistently normal ALT, with the highest prevalence in the high-normal subgroup [29], and Wong et al. reported F2 fibrosis in 37% of immunotolerant patients aged over 35 with high-normal ALT, compared with only 2% in those with low-normal ALT [30]. These findings, together with a European biopsy cohort in which 36% of ALT-normal CHB patients had significant fibrosis and reports of significant histologic alterations in untreated pediatric CHB cohorts [28,31,32], suggest that the presence of normal ALT should not be equated with histological quiescence—though it is important to note that these estimates derive from selected cohorts and may not be directly generalisable.

Non-invasive tools, particularly transient elastography, offer a practical means of identifying fibrosis in this population without the risks and sampling limitations of liver biopsy [33]. In the present study, LSM identified significant fibrosis in 18.4% of ALT-normal patients, underscoring the potential complementary value of routine elastography alongside standard biochemical monitoring. Current EASL guidelines recommend elastography or biopsy for HBeAg-positive patients with ALT 1–2× ULN, and support antiviral therapy for patients over 30 years of age even without formal fibrosis staging results [3]. Our findings are broadly aligned with this risk-stratified approach, though we acknowledge that the LSM threshold of 7.0 kPa used here as an operational cut-off for significant fibrosis carries known limitations, including susceptibility to overestimation in the setting of active necroinflammation.

### 4.4. Quantitative HBsAg as a Complementary Stratification Marker

In this cohort, higher quantitative HBsAg was independently associated with lower LSM in both linear and logistic regression analyses, and patients with HBsAg ≥ 25,000 IU/mL had significantly lower liver stiffness than those below this threshold. These observations are consistent with prior reports suggesting that very high HBsAg levels may identify a subset of HBeAg-positive patients with lower concurrent fibrosis burden [34,35]. A plausible biological basis—whereby extremely high antigen loads maintain deeper immune suppression and thereby limit fibrogenic activity—has been proposed in the mechanistic literature cited above, though this remains to be formally tested. Importantly, the clinical utility of any specific HBsAg threshold is likely to vary across populations due to differences in viral genotype, precore and basal core promoter mutation prevalence, and host immunogenetic background. The 25,000 IU/mL cut-off explored here should therefore be regarded as exploratory, and external validation in ethnically and geographically diverse cohorts will be required before it can inform clinical decision-making.

### 4.5. Clinical Implications

The principal clinical message of these findings is that ultra-high viral load should not, in isolation, be interpreted as evidence of a uniformly low-risk state or used as a sole basis for deferring further evaluation. The present data support a multi-parameter approach to risk stratification that integrates ALT, AST, quantitative HBsAg, HBV DNA, and non-invasive fibrosis assessment. Such an approach may improve identification of patients at risk of silent progression who would be missed by ALT-based criteria alone. It should be emphasised, however, that this study does not provide evidence regarding longitudinal outcomes—including cirrhosis development, treatment response, or HCC incidence—and the clinical implications outlined here are therefore inferential. Prospective studies with long-term follow-up, systematic histological confirmation, and genotype and mutation data will be needed to establish the prognostic and therapeutic significance of the heterogeneity described.

Histology remains the reference standard for fibrosis and necroinflammation assessment, and liver biopsy retains particular value in patients with discordant non-invasive findings, unexplained enzyme elevations, or diagnostic uncertainty around treatment timing. The use of elastography as the primary fibrosis measure in this study reflects the constraints of a retrospective dataset rather than a position that biopsy is dispensable; in clinical practice, non-invasive and invasive assessments are best regarded as complementary.

### 4.6. Limitations

Several limitations of this study warrant explicit acknowledgement. First, the retrospective single-centre design introduces the possibility of selection bias and limits generalisability to other populations or practice settings. Second, the analysis is cross-sectional at baseline; no longitudinal follow-up data are available, and causal inferences regarding disease progression cannot be drawn. Third, liver biopsy was not performed systematically, so fibrosis staging relies entirely on elastography-derived LSM, which may overestimate fibrosis during episodes of active hepatic inflammation. Fourth, HBV genotype distribution and precore/basal core promoter mutation data were unavailable, precluding a more complete characterisation of virological heterogeneity. Fifth, the duration of infection was unknown for most patients, so the observed association between age and fibrosis may partly reflect unmeasured infection duration. Finally, the HBsAg threshold of 25,000 IU/mL and other proposed cut-offs may not generalise across populations with different viral and host genetic backgrounds, and require prospective external validation.

## 5. Conclusions

In untreated HBeAg-positive CHB patients with ultra-high viral load (HBV DNA ≥ 7.0 log_10_ IU/mL), substantial baseline heterogeneity exists across biochemical, virological, and fibrosis-related domains. Using LSM ≥ 7.0 kPa as a non-invasive operational threshold for significant fibrosis, we identified a clinically meaningful proportion of patients with silent progression despite normal ALT. Age and AST were independently associated with significant fibrosis, while higher HBV DNA and HBsAg levels were associated with lower LSM-defined fibrosis burden in this cohort.

These findings support a risk-stratification strategy that integrates ALT, AST, quantitative HBsAg, HBV DNA, and LSM rather than relying on ALT alone. Quantitative HBsAg may provide complementary information for identifying lower-fibrosis phenotypes, but any threshold-based clinical use requires external validation across populations with different viral and host backgrounds. Prospective multicenter studies with longitudinal follow-up, genotype/mutation data, and histologic confirmation in selected cases are needed to confirm the prognostic and therapeutic implications.

## Figures and Tables

**Table 1:** Baseline characteristics of the study cohort.

Indicator	Overall (*n* = 413)	Male (*n* = 217)	Female (*n* = 196)	*p* Value
Age (years)	38.23 ± 9.60	38.14 ± 9.50	38.32 ± 9.75	0.870
HBV DNA [log_10_ (IU/mL)]	8.24 (8.00, 8.64)	8.25 (8.00, 8.65)	8.23 (8.00, 8.62)	0.728
HBsAg (IU/mL)	30,109.00 (9902.00, 55,855.00)	32,063.00 (9902.00, 55,855.00)	27,844.00 (9902.00, 55,855.00)	0.740
HBeAg (IU/mL)	1320.00 (887.00, 1597.00)	1411.00 (887.00, 1597.00)	1392.00 (887.00, 1597.00)	0.560
ALT (U/L)	59.00 (30.00, 123.00)	71.00 (30.00, 123.00)	49.00 (25.00, 71.00)	<0.001
AST (U/L)	41.00 (25.00, 71.00)	43.00 (25.00, 71.00)	36.00 (25.00, 71.00)	0.080
CAP (dB/m)	223.00 (195.00, 257.00)	231.00 (195.00, 257.00)	211.00 (195.00, 257.00)	0.005
LSM (kPa)	6.40 (5.10, 9.20)	6.50 (5.10, 9.20)	6.10 (5.10, 9.20)	0.040

Note: Age is expressed as mean ± standard deviation; other quantitative data are presented as median (interquartile range). HBV DNA values are log-transformed using base 10. ALT: Alanine aminotransferase; AST: Aspartate aminotransferase; CAP: Controlled Attenuation Parameter; HBeAg: Hepatitis B e antigen; HBsAg: Hepatitis B surface antigen; LSM: Liver stiffness measurement. Intergroup comparisons were performed using the Mann–Whitney U test. *p* < 0.05 was considered statistically significant.

**Table 2:** Multivariate analysis of independent predictors for LSM.

Variable	β Value	Standard Error	Lower 95% CI	Upper 95% CI	*p* Value
Constant term	−2.784	11.324	−24.979	19.410	0.806
Age (years)	0.133	0.052	0.031	0.235	0.011
Gender (Female)	−0.140	0.890	−1.885	1.605	0.875
ALT (U/L)	0.003	0.005	−0.006	0.012	0.537
AST (U/L)	0.011	0.006	−0.001	0.023	0.073
CAP (dB/m)	0.017	0.012	−0.006	0.039	0.151
log_10_HBsAg	−3.063	1.381	−5.769	−0.357	0.027
log_10_HBV DNA	1.842	1.177	−0.466	4.149	0.118
AFP (ng/mL)	0.005	0.018	−0.031	0.042	0.772

**Table 3:** Analysis of Independent Risk Factors for Significant Liver Fibrosis.

Variable	OR Value	95% CI Lower Limit	95% CI Upper Limit	*p* Value
Constant Term	72.995	1.216	4382.386	0.040
Age (years)	1.049	1.025	1.073	<0.001
Gender (female)	0.759	0.479	1.201	0.239
ALT (U/L)	1.000	0.998	1.003	0.760
AST (U/L)	1.005	1.001	1.009	0.010
CAP (dB/m)	1.005	0.999	1.010	0.090
log_10_HBsAg	0.585	0.369	0.927	0.023
log_10_HBV DNA	0.512	0.310	0.847	0.001
AFP (ng/mL)	1.000	0.999	1.001	0.813

**Table 4:** Two-dimensional Hierarchy of ALT and LSM (all four subgroups included).

ALT (U/L)	LSM (kPa)	Sample Size (*n*)	Percentage (%)
≤40	<7	115	81.560
≤40	≥7	26	18.440
>40	<7	130	47.794
>40	≥7	142	52.206

**Table 5:** Comparison of Liver Stiffness Measurement (LSM) based on HBsAg threshold (25,000 IU/mL).

Groups (HBsAg Stratification)	Sample Size (*n*)	Median LSM (IQR), kPa	*p* Value
HBsAg < 25,000 IU/mL	183	7.60 (5.70–12.40)	<0.001
HBsAg ≥ 25,000 IU/mL	230	6.00 (4.80–7.55)	<0.001

**Table 6:** Baseline characteristics of chronic hepatitis B patients with high viral load, stratified by cluster.

Characteristics	Cluster 1 (*n* = 130)	Cluster 2 (*n* = 283)
Age (years), mean ± SD	42.97 ± 10.18	35.37 ± 9.24
ALT (U/L), median (IQR)	113.50 (49.25–271.75)	49.00 (26.00–87.00)
LSM (kPa), median (IQR)	10.15 (6.78–17.57)	5.80 (4.80–6.90)
CAP (dB/m), median (IQR)	223.00 (198.25–253.75)	222.00 (193.00–258.50)
HBV DNA (log_10_ IU/mL), median (IQR)	7.849 (7.449–8.176)	8.471 (8.230–8.716)
HBsAg (log_10_ IU/mL), median (IQR)	3.738 (3.477–4.158)	4.663 (4.399–4.823)
ALT > 40 U/L, *n* (%)	111 (85.4)	161 (56.9)
LSM ≥ 7.0 kPa, *n* (%)	97 (74.6)	71 (25.1)
Silent progression *, *n* (%)	12 (9.2)	14 (4.9)

Note: * Silent progression refers to patients with ALT < 40 U/L and LSM ≥ 7.0 kPa.

## Data Availability

The data are part of an ongoing study.

## Supplementary Materials

The following supporting information can be downloaded at: https://www.mdpi.com/article/10.3390/jcm15062164/s1, Figure S1: Relationship plots of ALT, age, HBV DNA, and fibrosis-related markers in HBeAg-positive CHB patients with ultra-high viral load; Table S1: Subgroup Comparison Table Based on HBV DNA Stratification; Table S2: Subgroup Comparison by Age Stratification; Table S3: Comparison of subgroups stratified by ALT; Table S4: Comparison of subgroups stratified by LSM.

## Abbreviations

ALT, alanine aminotransferase; AST, aspartate aminotransferase; AFP, alpha-fetoprotein; CAP, controlled attenuation parameter; CHB, chronic hepatitis B; CI, confidence interval; HBeAg, hepatitis B e antigen; HBsAg, hepatitis B surface antigen; HBV, hepatitis B virus; HCC, hepatocellular carcinoma; IQR, interquartile range; LSM, liver stiffness measurement; OR, odds ratio; PCA, principal component analysis; SD, standard deviation; ULN, upper limit of normal.
