# Supplementary material for: Heterogeneity Analysis of HBeAg-Positive Chronic Hepatitis B Patients with Ultra-High Viral Load (HBV DNA ≥ 7.0 log10 IU/mL)
Source: J Clin Med. 2026 Mar 12;15(6):2164. doi: 10.3390/jcm15062164 (PMC13026527; doi:10.3390/jcm15062164)
Supplement: Supplementary file 1 [file jcm-15-02164-s001.zip › jcm-4144641-supplementary.pdf]

Supplementary Material

Note: Continuous variables are expressed as median [Q1, Q3], and categorical variables are expressed as n (%). Kruskal-Wallis tests were used for continuous variables, and chi-square tests were used for categorical variables.

Table S1 Subgroup Comparison Table Based on HBV DNA Stratification

Variable	<1e8 IU/mL (n=89)	1e8-1e9 IU/mL (n=297)	>=1e9 IU/mL (n=27)	P-value
Age (years)	41.00 [32.00, 47.00]	35.00 [31.00, 41.00]	37.00 [30.00, 48.00]	0.008
ALT (U/L)	98.00 [51.00, 185.00]	54.00 [27.00, 111.00]	46.00 [28.50, 121.50]	<0.001
AST (U/L)	64.00 [34.00, 125.00]	36.00 [24.00, 64.00]	38.00 [24.00, 63.50]	<0.001
HBsAg (IU/mL)	6643.00 [2763.00, 16320.00]	36767.00 [16371.00, 59954.00]	64357.00 [40436.00, 89646.00]	<0.001
HBeAg	712.00 [230.00, 1346.00]	1411.00 [1044.50, 1630.00]	1490.50 [1061.00, 1683.50]	<0.001
LSM (kPa)	8.70 [6.10, 13.20]	6.10 [5.00, 8.50]	6.50 [5.65, 8.65]	<0.001
CAP (dB/m)	223.00 [188.00, 254.00]	220.00 [194.00, 257.00]	242.00 [212.50, 279.50]	0.042
AFP (ng/mL)	5.18 [2.70, 9.45]	3.27 [2.22, 5.00]	2.88 [2.12, 4.94]	<0.001
AST/ALT ratio	0.71 [0.43, 0.96]	0.73 [0.57, 0.94]	0.77 [0.57, 1.05]	0.202
Family history, n (%)	84 (94.4%)	275 (92.6%)	26 (96.3%)	0.677
ALT >80 U/L, n (%)	47 (52.8%)	105 (35.4%)	12 (44.4%)	0.011

Table S2: Subgroup Comparison by Age Stratification

Variable	<30 y (n=70)	30-60 y (n=332)	>60 y (n=11)	P-value
ALT (U/L)	66.00 [37.25, 138.25]	57.00 [30.00, 119.25]	42.00 [25.50, 87.00]	0.256
AST (U/L)	44.00 [28.00, 81.50]	40.00 [25.00, 71.00]	36.00 [25.50, 59.00]	0.357
HBsAg (IU/mL)	31048.50 [12461.00, 50947.25]	32107.00 [9769.25, 58530.00]	15688.00 [2306.50, 23730.00]	0.043
HBeAg	1358.00 [1069.50, 1695.25]	1360.00 [712.00, 1620.00]	1150.50 [892.25, 1320.50]	0.234
HBV DNA (IU/mL)	171500000.00 [100000000.00, 388750000.00]	181000000.00 [100000000.00, 450000000.00]	102000000.00 [35950000.00, 127000000.00]	0.029
LSM (kPa)	5.90 [4.80, 7.38]	6.50 [5.10, 9.77]	7.00 [5.85, 12.75]	0.013
CAP (dB/m)	214.50 [184.25, 255.50]	224.50 [197.75, 257.25]	195.00 [185.00, 222.00]	0.050
AFP (ng/mL)	2.67 [1.86, 4.04]	3.51 [2.34, 6.11]	8.02 [4.25, 40.25]	<0.001
AST/ALT ratio	0.69 [0.52, 0.86]	0.73 [0.55, 0.96]	0.92 [0.76, 1.11]	0.197
Family history, n (%)	67 (95.7%)	307 (92.5%)	11 (100.0%)	0.410
ALT >80 U/L, n (%)	31 (44.3%)	130 (39.2%)	3 (27.3%)	0.505

Table S3 Comparison of subgroups stratified by ALT

Variable	<20 U/L (0-0.5 ULN) (n=37)	20-40 U/L (0.5-1 ULN) (n=103)	40-80 U/L (n=113)	>80 U/L (n=160)	P-value
Age (years)	37.00 [33.00, 46.00]	35.00 [32.00, 42.00]	36.00 [30.00, 45.00]	36.00 [31.00, 43.00]	0.697
AST (U/L)	18.00 [17.00, 21.00]	24.00 [20.00, 28.00]	38.00 [31.00, 48.00]	84.00 [62.00, 214.25]	<0.001
HBsAg (IU/mL)	52769.00 [25269.00, 74203.00]	49071.00 [25203.00, 63929.50]	28605.00 [10388.00, 51054.00]	14682.50 [5969.25, 44934.25]	<0.001
HBeAg	1570.00 [1200.00, 1715.00]	1484.50 [1320.00, 1630.00]	1343.50 [945.75, 1621.00]	1151.00 [390.00, 1583.50]	0.007
HBV DNA (IU/mL)	347000000.00 [153000000.00, 564000000.00]	323000000.00 [170000000.00, 541000000.00]	170000000.00 [89200000.00, 400000000.00]	170000000.00 [69900000.00, 328500000.00]	<0.001
LSM (kPa)	5.50 [4.40, 6.10]	5.50 [4.60, 6.65]	6.40 [5.30, 9.60]	8.15 [6.20, 13.20]	<0.001
CAP (dB/m)	200.00 [189.00, 236.00]	233.00 [199.50, 269.00]	223.00 [200.00, 256.00]	219.00 [188.75, 252.25]	0.012
AFP (ng/mL)	2.88 [1.63, 4.20]	2.67 [2.06, 4.07]	3.61 [2.29, 5.71]	4.40 [2.67, 8.46]	<0.001
AST/ALT ratio	1.17 [1.00, 1.44]	0.88 [0.77, 1.05]	0.67 [0.55, 0.78]	0.57 [0.41, 0.74]	<0.001
Family history, n (%)	33 (89.2%)	98 (95.1%)	107 (94.7%)	147 (91.9%)	0.494
ALT >80 U/L, n (%)	0 (0.0%)	5 (4.9%)	2 (1.8%)	157 (98.1%)	<0.001

Table S4 Comparison of subgroups stratified by LSM

Variable	LSM <7 kPa (n=245)	7-9.5 kPa (n=67)	>=9.5 kPa (n=101)	P-value
Age (years)	35.00 [30.00, 41.00]	36.00 [31.00, 44.00]	41.00 [34.00, 48.00]	<0.001
ALT (U/L)	44.00 [25.00, 79.00]	99.00 [56.00, 171.50]	93.00 [47.00, 188.00]	<0.001
AST (U/L)	33.00 [23.00, 52.00]	63.00 [38.00, 107.00]	64.00 [36.00, 162.00]	<0.001
HBsAg (IU/mL)	37284.00 [16553.00, 62402.00]	20458.00 [6714.00, 55268.00]	10684.00 [4006.00, 41942.00]	<0.001
HBeAg	1431.00 [1217.00, 1649.00]	1344.00 [623.50, 1592.00]	810.00 [254.00, 1530.00]	<0.001
HBV DNA (IU/mL)	237000000.00 [130000000.00, 492000000.00]	170000000.00 [44900000.00, 263000000.00]	160000000.00 [63000000.00, 400000000.00]	<0.001
CAP (dB/m)	220.00 [191.00, 256.00]	222.00 [198.00, 255.00]	228.00 [200.00, 261.00]	0.566
AFP (ng/mL)	2.82 [1.98, 4.29]	3.67 [2.76, 6.00]	6.70 [3.30, 21.14]	<0.001
AST/ALT ratio	0.75 [0.57, 0.96]	0.63 [0.44, 0.86]	0.70 [0.55, 0.98]	0.039
Family history, n (%)	230 (93.9%)	63 (94.0%)	92 (91.1%)	0.618
ALT >80 U/L, n (%)	63 (25.7%)	46 (68.7%)	55 (54.5%)	<0.001

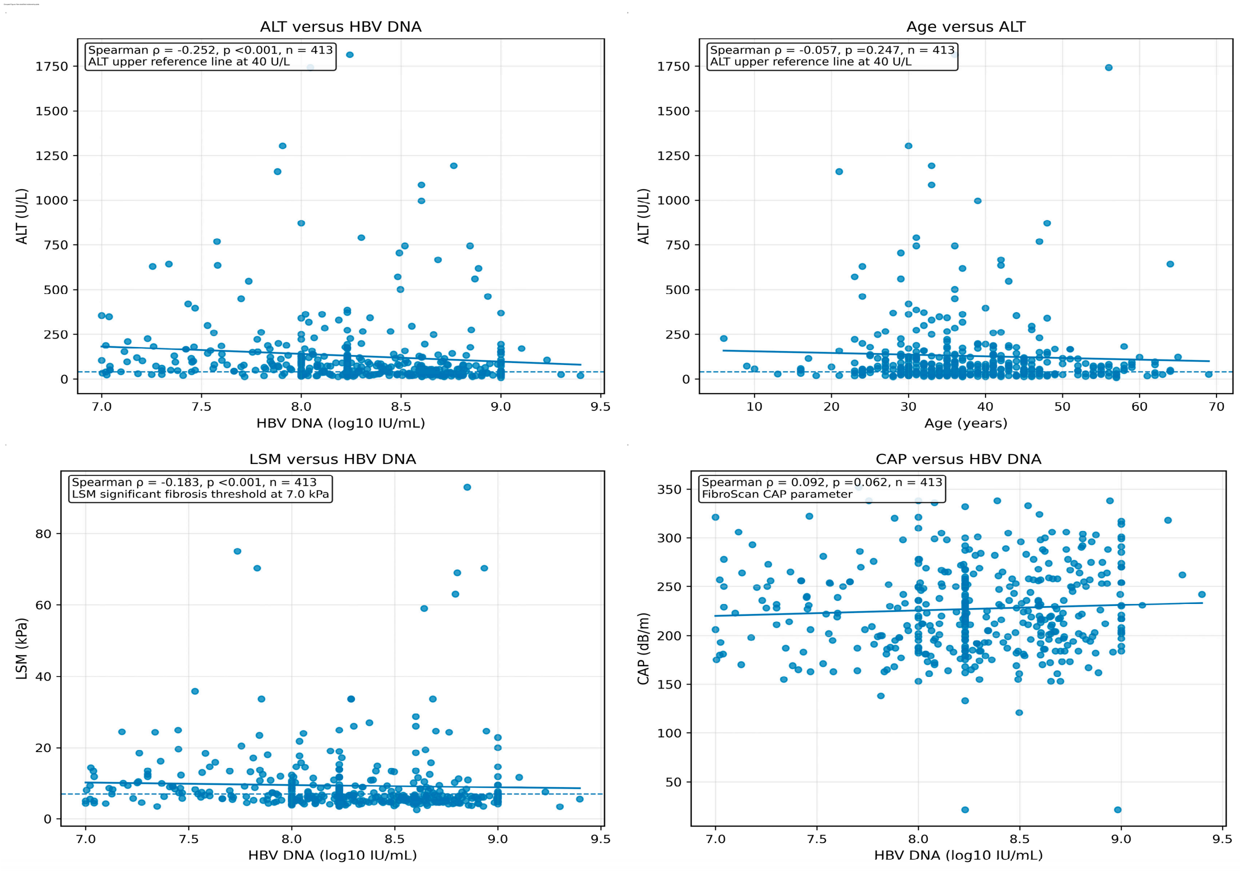

Supplementary Figure S1. Relationship plots of ALT, age, HBV DNA, and fibrosis-related markers in HBeAg-positive CHB patients with ultra-high viral load
